# Changes in sedation management in German intensive care units between 2002 and 2006: a national follow-up survey

**DOI:** 10.1186/cc6189

**Published:** 2007-12-06

**Authors:** Jörg Martin, Martin Franck, Stefan Sigel, Manfred Weiss, Claudia Spies

**Affiliations:** 1 Department of Anesthesiology, Intensive Care Medicine and Pain Therapy, Hospital am Eichert, Eichertstraße 3, 73035 Göppingen, Germany; 2 Department of Anesthesiology and Intensive Care Medicine, Charité-Universitätsmedizin Berlin, Campus Mitte, Charitéplatz 1, 10117 Berlin, Germany; 3 Department of Anesthesiology, Universitätsklinikum Ulm, Steinhövelstraße 9, 89075 Ulm, Germany

## Abstract

**Background:**

The aim of this study, conducted in 2006, was to find out whether changes in sedation management in German intensive care units took place in comparison with our survey from 2002.

**Methods:**

We conducted a follow-up survey with a descriptive and comparative cross-sectional multi-center design. A postal survey was sent between January and May 2006, up to four times, to the same 269 hospitals that participated in our first survey in 2002. The same questionnaire as in 2002 was used with a few additional questions.

**Results:**

Two hundred fourteen (82%) hospitals replied. Sixty-seven percent of the hospitals carried out changes in sedation management since the 2002 survey. Reasons for changes were published literature (46%), national guidelines (29%), and scientific lectures (32%). Sedation protocols (8% versus 52%) and a sedation scale (21% versus 46%) were used significantly more frequently. During sedation periods of up to 24 hours, significantly less midazolam was used (46% versus 35%). In comparison to 2002, sufentanil and epidural analgesia were used much more frequently in all phases of sedation, and fentanyl more rarely. For periods of greater than 72 hours, remifentanil was used more often. A daily sedation break was introduced by 34% of the hospitals, and a pain scale by 21%.

**Conclusion:**

The increased implementation of protocols and scoring systems for the measurement of sedation depth and analgesia, a daily sedation break, and the use of more short-acting analgesics and sedatives account for more patient-oriented analgesia and sedation in 2006 compared with 2002.

## Introduction

Most mechanically ventilated patients require analgesia and sedation. Adequate analgesia and sedation should ensure that the patient can receive intensive medical care without undue stress or pain [1]. As with insufficient sedation, excessively deep sedation also may lead to increased morbidity, increased costs, and a prolonged stay in the intensive care unit (ICU) [2-4]. Recent advances with drugs that are more controllable, better ventilation techniques and sedation strategies, and the use of scoring systems and sedation protocols enable optimization of sedation [1,5,6]. However, the optimal sedation strategy remains a controversial issue at present. Despite controversies, a shift from deeper to lighter sedation, thereby maintaining the normal circadian rhythm, is emerging within the published literature [1,5,7].

Apart from sedation, emphasis should be placed on adequate monitoring of analgesia. Whipple and colleagues [8] have shown that 70% of patients admitted to an ICU recollected suffering pain. Regular review of pain scores followed by appropriate therapy leads to a reduction in morbidity and a reduction in duration of mechanical ventilation [9].

Soliman and colleagues [10] have shown that sedation practice and the use of differing scoring systems and guidelines are quite different between countries. In 2002, a national survey of analgesia and sedation practice in German ICUs was undertaken [11]. The goal of the present study was to find out whether new trends or significant changes in ICU analgesia and sedation practice among mechanically ventilated patients in ICUs have emerged since the last survey [11] and the publication of national guidelines for analgesia and sedation practice in Germany in 2005 [12]. The major recommendations of the guidelines consist of the application of scores for analgesia and sedation, with a patient-oriented depth of sedation, implementation of written sedation protocols, application of short-acting drugs, and use of regional anesthesia techniques.

## Materials and methods

This study is a descriptive, comparative, multi-center follow-up study to the survey performed in 2002 [11]. The same address database was used as in the first study. In total, 254 general hospitals and 15 university hospitals were contacted. All ICUs were run by an anesthesiological department and include surgical and interdisciplinary adult ICUs. The survey was submitted to the chairmen of the departments up to four times in the period January to May 2006. The response rate was 82% and most of the forms were filled out by the head of the ICU. The questionnaire corresponded to the first questionnaire, although a few questions were added.

### The questionnaire

A modified version of the questionnaire used in 2002 [11] was used. Questions were added relating to the use of pain scores [9], daily sedation breaks [2,13], and the reasons for any changes in sedation practice. Since no department used a validated instrument to score for delirium in the previous survey, we did not ask about delirium in the present questionnaire.

#### Part 1: General data

General data included size and type of ICU, number of beds, case mix index, number of days in the ICU, average length of stay, number of mechanically ventilated patients per year, and average duration of ventilation.

#### Part 2: Data relating to analgesia and sedation

These data included changes accomplished in sedation management since 2002, use of scores for pain severity and sedation depth, daily sedation break, implementation of written protocols, reasons for choice of medications used, and use of muscle relaxants.

#### Part 3: Questions relating to changes in analgesia and sedation practice occurring since the last survey

These questions focused on reasons for any potential changes that had occurred in the interim period between this survey and the 2002 survey.

#### Part 4: Pharmacological and regional analgesia techniques for analgesia and sedation

As was the case with the first survey, the sedation periods were divided into the following groups according to the American [14] and German [12] guidelines: sedation up to 24 hours, 24 to 72 hours, greater than 72 hours, and analgesia and sedation during weaning from mechanical ventilation.

### Statistics

Data were collected in a Microsoft Access 2002 database and analyzed with Microsoft Excel 2002 (Microsoft Corporation, Redmond, WA, USA) and SPSS for Windows (version 13.0; SPSS Inc., Chicago, IL, USA). Responses to part 2 of the questionnaire were given as 'yes' or 'no'. Responses to parts 3 and 4 may include more than one answer. Univariate statistical analyses were carried out depending on the scaling of the data, using either the Mann-Whitney *U *test or the chi-square test. Analyses were assigned as exploratory. Therefore, no multiple adjustments were carried out. Statistical significance (in the sense of exploratory analyses) was set at a *p *value of greater than 0.05.

## Results

The response rate was 82% (214 out of 261). More than 90% of the ICUs that responded in 2002 also participated in this survey. At this rate of response, non-responder bias is insignificant [15]. There was no significant difference to the 2002 survey response rate (82%). All data were entered into the analyses. In regard to general data (Table 1), there were significant differences in the number of ICU beds in university hospitals and in general hospitals (9 versus 10) and also in the number of patients treated annually (9,330 versus 1,000).

**Table 1:** General data of the surveyed intensive care units in 2002 and 2006

	2002 Median (minimum, maximum)	2006 Median (minimum, maximum)	*P *value
Intensive care beds (university hospitals)	14 (8, 24)	24 (10, 32)	0.09
Intensive care beds (general hospitals)	9 (2, 27)	10 (4, 40)	0.02
Patients per year (university hospitals)	1,481 (517, 2,500)	2,000 (700, 2,860)	0.2
Patients per year (general hospitals)	930 (120, 5,297)	1,000 (120, 4,578)	0.03
Nursing care days (university hospitals)	4,950 (2,000, 7,900)	5,000 (3,000, 13,900)	0.5
Nursing care days (general hospitals)	2,704 (400, 7,830)	3,000 (250, 15,462)	0.06

The Mann-Whitney *U *test was used to calculate *p *values.

### Process data

Whereas only 8% of hospitals used a sedation scale in the first survey, this number rose to 51% in the present survey. We do not know how these scales were directed at the individual patient level in terms of comparing target and actual sedation levels. The Ramsay sedation scale (RSS) [16] was used almost exclusively, and only one hospital referred to use of the Richmond agitation sedation scale (RASS) [17]. Only numeric rating scales and visual analogue scales were used as a pain scale. Furthermore, sedation protocols were employed significantly more often (by 46% of respondents compared with 21% in the first survey). Oral procedure instructions (departmental consensus) were introduced in 93% of the surveyed hospitals, and this differed significantly from the first survey. Eighty-eight percent of the hospitals endeavored to maintain a day-night rhythm (Table 2).

**Table 2:** Sedation practices in 2002 and 2006

	2002 (*n *= 220)	2006 (*n *= 214)	*P *value
	Percentage (95% CI)		

Sedation score	8 (-4 to 20)	51 (41 to 60)	<0.001
Sedation protocol	21 (9 to 32)	46 (36 to 56)	<0.001
Medication selection according to sedation duration	92 (88 to 96)	93 (89 to 97)	0.5
Oral procedure instructions	44 (33 to 53)	93 (89 to 97)	<0.001
Day-night rhythm	81 (75 to 87)	88 (84 to 94)	<0.001
Consideration of costs affecting drug selection	62 (54 to 70)	55 (46 to 64)	0.4

The chi-square test was used to calculate *p *values. CI, confidence interval; n, number of positive answers.

### Changes in analgesia and sedation practice

Sixty-seven percent (95% confidence interval: 37% to 97%) of hospitals changed analgesia and sedation practice in the years 2002 to 2006. Reasons given for these changes ranged from the publication of the S2e guidelines (29%) to educational lectures (32%). The most frequently quoted reason resulting in a change in analgesia and sedation policy was studying current literature (46%). The reasons for the changes are summarized in Table 3. Pain scoring, although not surveyed in 2002, was found to be in use in 21% of cases. Thirty-four percent of respondents had introduced a daily sedation break (Table 4).

**Table 3:** Reasons given for changes in sedation management since the last survey (2002)

Changes in sedation management	Percentage (95% CI) *n *= 214
Resulting from S2e guidelines	29 (18 to 40)
Resulting from change in department leadership	10 (-3 to 23)
Following lectures	32 (21 to 42)
Following literature	46 (36 to 56)
Other	13 (-6 to 32)

CI, confidence interval; n, number of positive answers.

**Table 4:** Changes accomplished in sedation management since 2002

Changes	Percentage (95% CI) *n *= 214
Sedatives changed	33 (22 to 44)
Analgesia changed	31 (20 to 42)
Regional techniques introduced	19 (7 to 31)
Pain scoring introduced	21 (9 to 33)
Daily sedation break	34 (23 to 45)
Sedation protocol implemented	23 (11 to 35)
Other	4 (-9 to 17)

CI, confidence interval; n, number of positive answers.

### Medications and techniques

Only medications or interventions used by more than 5% of the respondents in any of the time periods were considered.

#### Sedatives

Propofol was used most frequently for sedation up to 24 hours (83%). Midazolam was used significantly less frequently during this period than in the 2002 survey (46% versus 35%). In regard to periods of greater than 72 hours and weaning, use of sedatives did not change (Tables 5 and 6).

**Table 5:** Comparison of the techniques and agents used for analgesia and sedation in 2002 and 2006

	Less than 24 hours			24 to 72 hours		
	Percentage (95% CI)		*P *value	Percentage (95% CI)		*P *value

Year	2002 *n *= 220	2006 *n *= 214		2002 *n *= 220	2006 *n *= 214	
Midazolam	45.9 (36.1 to 55.6)	34.6 (23.7 to 45.4)	<0.001	77.3 (71.0 to 83.6)	62.2 (53.9 to 70.4)	<0.001
Propofol	81.4 (75.7 to 87.1)	82.7 (77.1 to 88.3)	0.6	55,9 (47.2 to 64.7)	67.3 (59.6 to 74.9)	<0.001
Remifentanil	5.9 (-6.9 to 18.7)	16.8 (4.6 to 29.0)	<0.001	2.3 (-10.8 to 15.3)	7.9 (-4.9 to 20.8)	<0.002
Fentanyl	40.0 (29.8 to 50.3)	27.1 (15.7 to 38.5)	<0.001	55.9 (47.1 to 64.7)	40.7 (30.3 to 51.0)	<0.001
Sufentanil	35.0 (24.4 to 45.7)	41.6 (31.4 to 51.8)	<0.05	47.7 (38.2 to 57.3)	58.4 (49.8 to 67.1)	<0.002
Piritramide	38.2 (27.8 to 48.6)	35.1 (24.3 to 45.8)	0.3	15.5 (3.3 to 27.6)	16.8 (4.6 to 29.0)	0.6
Morphine	8.6 (-4.0 to 21.3)	7.5 (-5.4 to 20.4)	0.5	4.5 (-8.4 to 17.5)	7.9 (-4.9 to 20.8)	0.06
PCA	25.5 (14.0 to 36.9)	28.5 (17.2 to 39.8)	0.3	15.5 (3.3 to 27.6)	21.5 (9.6 to 33.4)	<0.001
Ketamine (S)	6.8 (-5.9 to 19.6)	14.5 (2.1 to 26.9)	0.001	20.0 (8.2 to 31.8)	25.7 (14.2 to 37.2)	<0.001
Clonidine	35.9 (25.3 to 46.5)	33.6 (22.7 to 44.6)	0.5	48.2 (38.7 to 57.7)	49.5 (40.0 to 59.1)	0.7
NSAIDs	26.8 (15.6 to 38.2)	23.8 (12.1 to 35.5)	0.3	13.2 (0.8 to 25.5)	16.8 (4.6 to 29.0)	0.1
NMBAs	3.6 (-9.3 to 16.6)	0.0	0.5	0.0	0.0	n.c.
PNB	15.5 (3.3 to 27.6)	23.8 (12.1 to 35.5)	0.004	12.7 (0.4 to 25.1)	22.0 (10.1 to 33.8)	<0.001
PCEA	7.3 (-5.5 to 20.0)	11.7 (-0.9 to 24.3)	0.04	5.9 (-6.9 to 18.7)	9.8 (-2.9 to 22.5)	0.05
Epidural	68.2 (60.8 to 75.7)	72.0 (64.9 to 79.1)	0.5	59.1 (50.7 to 67.6)	73.8 (67.0 to 80.7)	<0.001

The chi-square test was used to calculate *p *values. CI, confidence interval; n, number of positive answers; n.c., not calculated; NMBAs, neuromuscular blocking agents; NSAIDs, non-steroidal anti-inflammatory drugs; PCA, patient-controlled analgesia; PCEA, patient-controlled epidural analgesia; PNB, peripheral nerve block.

**Table 6:** Comparison of the techniques and agents used for analgesia and sedation in 2002 and 2006

	More than 72 hours			Weaning		
	Percentage (95% CI)		*P *value	Percentage (95% CI)		*P *value

Year	2002 *n *= 220	2006 *n *= 214		2002 *n *= 220	2006 *n *= 214	
Midazolam	90.5 (86.4 to 94.5)	92.1 (88.3 to 95.8)	0.4	34.1 (23.4 to 44.8)	32.2 (21.2 to 43.3)	0.06
Propofol	26.4 (15.0 to 37.7)	22.9 (11.1 to 34.7)	0.2	72.3 (65.3 to 79.3)	75.2 (68.6 to 81.9)	0.3
Remifentanil	1.4 (-11.8 to 14.5)	3.7 (-9.4 to 16.9)	0.06	5.9 (-6.9 to 18.7)	15.0 (2.6 to 27.3)	<0.001
Fentanyl	65.0 (57.2 to 72.8)	53.7 (44.6 to 62.9)	<0.001	30.0 (19.0 to 41.0)	21.5 (9.6 to 33.4)	0.002
Sufentanil	43.6 (33.7 to 3.5)	51.4 (42.1 to 0.7)	0.02	41.8 (31.8 to 51.9)	49.5 (40.0 to 9.1)	0.02
Piritramide	9.1 (-3.5 to 21.7)	7.9 (-4.9 to 20.8)	0.5	25.5 (14.1 to 36.9)	24.3 (12.6 to 36.0)	0.7
Morphine	7.3 (-5.5 to 20.0)	9.8 (-2.9 to 22.5)	0.2	8.6 (-4.0 to 21.3)	9.4 (-3.4 to 22.1)	0.7
PCA	9.5 (-3.0 to 22.1)	7.0 (-5.9 to 19.9)	0.5	(-0.1 to 24.7)	12.2 (-0.4 to 24.7)	0.9
Ketamine (S)	19.1 (13.6 to 36.4)	19.6 (7.6 to 31.6)	0.7	5.0 (-7.9 to 17.9)	4.2 (-8.9 to 17.3)	0.7
Clonidine	56.4 (47.7 to 65.1)	52.8 (43.6 to 62.0)	0.3	62.7 (54.6 to 70.8)	58.9 (50.3 to 67.5)	0.5
NSAIDs	10.5 (-2.1 to 23.0)	10.3 (-2.4 to 22.9)	0.9	14.1 (1.8 to 26.3)	19.2 (7.1 to 31.2)	0.06
NMBAs	0.0	n.c.	n.c.	0.0	n.c.	n.c.
PNB	10.0 (-2.5 to 22.5)	14.5 (2.1 to 26.9)	0.6	7.7 (-5.0 to 20.4)	13.6 (1.1 to 26.0)	0.01
PCEA	4.1 (-8.9 to 17.0)	3.7 (-9.4 to 16.9)	0.8	4.6 (-8.4 to 17.5)	5.6 (-7.4 to 18.6)	0.5
Epidural	45.9 (36.2 to 5.6)	56.5 (47.7 to 5.4)	<0.005	42.3 (32.2 to 52.3)	52.8 (43.6 to 62.0)	<0.005

The chi-square test was used to calculate *p *values. CI, confidence interval; n, number of positive answers; n.c., not calculated; NMBAs, neuromuscular blocking agents; NSAIDs, non-steroidal anti-inflammatory drugs; PCA, patient-controlled analgesia; PCEA, patient-controlled epidural analgesia; PNB, peripheral nerve block.

#### Analgesics

Fentanyl was applied less frequently in all phases of sedation compared with the first survey. In the present survey, sufentanil was used significantly more often in all stages. For all periods of longer than 24 hours, remifentanil was more commonly ordered, with the greatest increases in the phases of 24 to 72 hours (6% versus 15%) and during weaning (2% versus 8%). The rates of application of morphine, piritramide, or non-steroidal anti-inflammatory drugs were comparable (Tables 5 and 6).

#### Adjuvant techniques for analgesia and sedation

With regard to the use of clonidine, there was no difference compared with the 2002 survey. Ketamine (S) was significantly more commonly used in the interim, particularly in the case of long-term sedation greater than 72 hours (7% versus 15%) and also during weaning (20% versus 26%). Patient-controlled analgesia techniques are now significantly more widely applied during weaning than in 2002 (16% versus 22%). Similar to the earlier survey, neuromuscular blockade was not performed regularly during any of the sedation phases (Tables 5 and 6).

#### Regional analgesia techniques

Epidural analgesia was observed more frequently in all stages of sedation compared with the preliminary investigation. Peripheral nerve blocks were also performed significantly more often for stays of greater than 72 hours and during weaning. Patient-controlled epidural analgesia was applied more often during short-term sedation periods (less than 24 hours) in the 2006 survey than in that of 2002 (Tables 5 and 6).

## Discussion

The goal of the present survey was to clarify whether a change has taken place in analgesia and sedation practice since the last survey in 2002 and after the publication of national practice guidelines on the subject [12]. Underlying the same questionnaire as in 2002, we are able to compare and contrast our findings. The most striking results are the significantly increased use of sedation scales and written sedation protocols.

The RSS [16] was the scale most frequently used in the present survey, with only one institution employing the RASS [17]. In recent reviews, Fraser and Rieker [18,19] demonstrated that only the consistent use of a validated sedation scale led to a reduction in the duration of mechanical ventilation, as well as reduced length of ICU stay. Written sedation guidelines were used significantly more often in the present survey. Employment of sedation guidelines enables reductions in both duration of mechanical ventilation and use of intensive care time [20], with consequent savings in medical costs [21]. However, as shown by Elliott and colleagues [22] and Andrews and colleagues [23], these positive changes are achieved only with sufficient training of the personnel involved and with adaptation of the guidelines to the local hospital environment. Meanwhile, a daily sedation break, as suggested by Kress and coworkers [2], is currently employed in one third of our surveyed hospitals. In addition, pain monitoring is increasingly performed. Chanques and colleagues [9] have pointed out that by monitoring and optimizing pain management, the number of nosocomial infections and the length of mechanical ventilation can be reduced. The use of analgesia and sedation scales, as well as the use of written guidelines, should result in avoidance of oversedation of patients, as described by Martin and colleagues [24].

As with the Danish follow-up questionnaire on sedation and analgesia in the ICU [25], a trend is being observed in Germany toward the application of analgesics and sedatives that are more controllable. For sedation periods of less than 72 hours, midazolam is used significantly less frequently than in 2002. Carson and colleagues [26] have shown the more favorable pharmacokinetics of propofol versus benzodiazepines. In terms of analgesics, the same development emerges. Fentanyl is used less frequently in all stages of sedation, but sufentanil is increasingly used. Remifentanil was applied more often for sedation periods of up to 24 hours, sedation for less than 72 hours, and during weaning. Breen and colleagues [27] and Muellejans and colleagues [28] have shown that using remifentanil in that way leads to a significantly shortened ventilation time compared with a control group. Ketamine (S) was more frequently used in the phases up to 24 hours and from 24 to 72 hours than in 2002. However, as has been shown by Ostermann and colleagues [29], there has been very little investigation of long-term use of ketamine sedation. Nevertheless, an important reason favoring the use of ketamine is the absence of a significant effect on bowel motility [30].

Epidural analgesia was performed more frequently in the phases of greater than 72 hours and during weaning than in the survey of 2002. As has been shown by Brodner and colleagues [31] and in the meta-analysis by Rodgers and colleagues [32], the perioperative use of epidural analgesia leads to both shortening of intensive care stay as well as lowering the incidence of cardiac and pulmonary complications. Thus, this approach of epidural analgesia is recommended in the German guidelines on analgesia and sedation in the ICU [12].

In total, two thirds (67%) of those taking part in the present survey reported changes in analgesia and sedation management since the last inquiry. Changes were induced by recent literature, scientific lectures, publication of national guidelines in 2005 [12], and a change in departmental leadership. Nevertheless, this current survey merely demonstrates that knowledge regarding analgesia and sedation policy has changed. Whether a real change in practice has occurred at the patient's bedside cannot be stated with certainty. In the implementation of guidelines in health care, large barriers to change exist [33], and so all parties of interest must be involved and motivated in order to effect changes in the daily treatment routine [34]. To evaluate actual analgesia and sedation practice, patient-centered investigations similar to those of Martin and colleagues [24] and Payen and colleagues [35] are needed.

## Conclusion

These results show that changes in analgesia and sedation practice among intensive care patients have taken place by the time of this survey, which was conducted 4 years after the initial investigation and 1 year after the appearance of national guidelines. In summary, the increased use of scoring systems for pain severity and sedation depth, a daily sedation break, the implementation of protocols, and the use of more short-acting medications for analgesia and sedation conform to the guidelines on patient-oriented analgesia and sedation. Although we see some areas of improvement, further efforts are necessary to reach widespread implementation of the analgesia and sedation guidelines into practice. Further patient-centered studies are needed to determine the extent to which these changes translate into performance at the patient's bedside. As wide variations in sedation practice exist internationally [10], further national investigations are needed to improve national sedation and analgesia policies, and, in the long run, to yield international consensus.

## Key messages

• According to our 2006 follow-up survey of German intensive care units, the following changes have occurred since 2002:

∘ Increased use of scoring systems for pain severity and sedation depth.

∘ Use of more short-acting medications for analgesia and sedation.

∘ A trend toward conformity with the national guidelines on patient-oriented analgesia and sedation.

• Also according to our follow-up survey, a daily sedation break is currently employed in only 34% of the hospitals.

## Abbreviations

ICU = intensive care unit; RASS = Richmond agitation sedation scale; RSS = Ramsay sedation scale.

## Competing interests

JM has been employed by GlaxoSmithKline (Uxbridge, Middlesex, UK) and B. Braun Melsungen AG (Melsungen, Germany) to present educational talks on therapy for analgesia and sedation and economics. The other authors declare that they have no competing or financial interests.

## Authors' contributions

JM made substantial contributions to the conception, design, analysis and interpretation of data. MF was involved in drafting the article and revising it critically for important intellectual content. SS performed the acquisition, analysis and interpretation of data. MW participated in the design and coordination and helped to draft the manuscript. CS made substantial contributions to the conception, design, analysis and interpretation of data.

All authors read and approved the final manuscript.
